# The Correlation Between Gender and Accessory Pathways

**DOI:** 10.7759/cureus.14746

**Published:** 2021-04-29

**Authors:** Hussein Rabah, Zaynab Khalaf, Rima Chaddad, Hassan Kazem, Bassam Ahmad, Hassan Mansour, Mohammad Saleh, Mohammad Boushnak, Mohamad K Moussa, Ali Rabah

**Affiliations:** 1 Internal Medicine, Staten Island University Hospital, Northwell Health, New York, USA; 2 Endocrinology, Diabetes and Metabolism, Lebanese University, Faculty of Medical Sciences, Al Hadath, LBN; 3 Interventional Cardiology, Lebanese University, Faculty of Medicine, Al Hadath, LBN; 4 Electrophysiology, Beirut Cardiac Institute, Beirut, LBN; 5 Cardiology, Lebanese University, Faculty of Medical Sciences, Al Hadath, LBN; 6 Internal Medicine, Lebanese University, Faculty of Medicine, Al Hadath, LBN; 7 Orthopedics and Traumatology, Lebanese University, Faculty of Medicine, Al Hadath, LBN; 8 Orthopedic Surgery, Lebanese University, Faculty of Medical Sciences, Al Hadath, LBN

**Keywords:** accessory pathways, manifest, gender, anatomical locations, concealed

## Abstract

Background

Accessory pathways (APs) are muscular bundles capable of rapid conduction between atria and ventricles. They can be located anywhere along the atrioventricular groove or septum. The etiology of such pathways is generally unknown. This study aims to evaluate the correlation between gender, AP location, and clinical presentation.

Methods

This is a retrospective study of 139 patients who underwent radiofrequency ablations for newly diagnosed accessory pathways between years 2010 and 2016. Information extracted from the medical records included: age at the time of diagnosis, gender, characteristics, and anatomical location of the accessory pathways.

Results

A total of 139 patients with AP were enrolled in the study. The mean age of diagnosis was 32.2 ± 13.5 years. With regards to gender, APs were more common among men (p-value 0.04). Males were predominant in both the right and left AP groups (p-value 0.025), although, overall, most of the AP were left located. Also, males were more commonly diagnosed with right posteroseptal (RPS) accessory pathways while females with left lateral (LL) pathways. Concerning the clinical presentation, the manifest form was more frequent than concealed. Males were prevalent in both groups (p-value 0.38).

Conclusion

Gender components might have a role in the pathogenesis of AP formation.

## Introduction

In the normal heart, the atria and ventricles are electrically isolated, with the conduction of electrical impulses from the atria to the ventricle occurring via the atrioventricular node. Patients with preexcitation syndrome have additional pathways that allow electrical continuity between atria and ventricles, thereby bypassing the atrioventricular (AV) node altogether [1]. Those additional pathways, known as accessory pathways (APs), are congenital in origin and result from the incomplete resorption of the myocardial syncytium at the fibrosis annulus during fetal development [2]. Conduction via the accessory pathways is faster than that via the AV node, and impulses from the atria reach the ventricles sooner than expected. This might predispose the patients to fatal ventricular arrhythmias [1].

The successful introduction of radiofrequency catheter ablation (RFCA) of the atrioventricular junction in 1982 stimulated interest in the nonsurgical ablation of atrioventricular accessory pathways [3-5]. Nowadays, this procedure provides a cure with minimal complication rates for such patients [6-7].

There are relatively few studies in the literature systematically describing the effect of gender on the location and manifestation of AP.

## Materials and methods

Study design

This was a secondary analysis of data from the Division of Cardiac Electrophysiology, Beirut Cardiac Institute, Lebanon. All patients newly diagnosed with accessory pathways between the years 2010 and 2016 were enrolled in the study. A total of 139 medical files were reviewed.

Information extracted from the medical records included age at the time of diagnosis, gender, characteristics, and anatomical locations of the accessory pathways.

APs were classified into 12 anatomical locations: left anteroseptal (LAS), left anterolateral (LAL), left lateral (LL), left posterolateral (LPL), left posterior (LP), left posteroseptal (LPS), right posteroseptal (RPS), right posterior (RP), right posterolateral (RPL), right lateral (RL), right anterolateral (RAL), and right anteroseptal (RAS) and into concealed or manifest based on their clinical manifestation.

Patients were divided into three age groups based on age at the time of diagnosis: at the age of 29 or younger, between ages of 30 and 49, and patients at age 50 and above.

Statistical analysis

Data were entered anonymously to ensure patient confidentiality and analyzed using the Statistical Package for the Social Sciences (SPSS) V26 (IBM Corp., Armonk, NY) to test the statistical significance among variables. The variables included in the analysis were age, gender, clinical characteristics, and locations of accessory pathways. A p-value less than 0.05 was considered statistically significant.

## Results

The age of patients at the time of diagnosis ranged between 10 and 70 years. Sixty-nine (50%) patients were diagnosed before or at the age of 29, 55 (39.5%) were diagnosed between 30 and 49 years, and the rest were diagnosed at age 50 or above. The mean age of diagnosis was 32.2 ± 13.5 years. Ninety-seven (69.8%) patients were males and 42 (30.2%) were females (p-value 0.04) (Table 1).

**Table 1:** The distribution of patients diagnosed with accessory pathways according to their age and gender (n=139)

Sex	Age group (y)	No. of patients (%)
Male	<29	50 (51%)
	30-49	35 (36.5%)
	>50	12 (12.5%)
Total		97 (100%)
Female	<29	19 (45.5%)
	30-49	20 (47.5%)
	>50	3 (7%)
Total		42 (100%)

**Figure 1:**
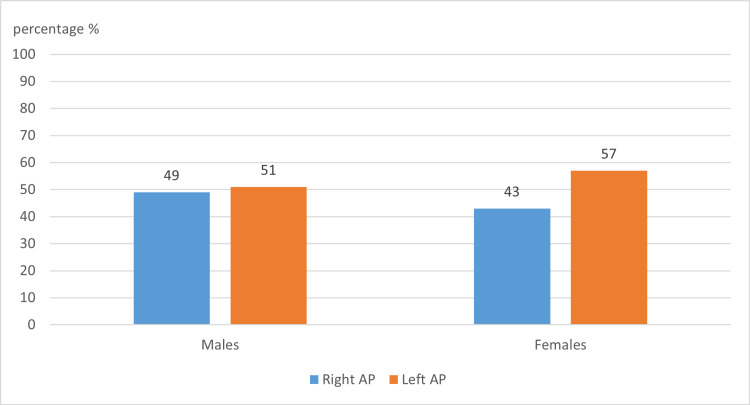
The distribution of right and left accessory pathways according to gender

Of the 139 records analyzed, 73 (52.5%) patients were diagnosed with left accessory pathways and 66 (47.5%) with right pathways. Further correlation with gender showed a statistically significant difference among pathway locations between males and females (p-value 0.025). Fifty-one percent of males and 57% of females had a left accessory pathway (Figure 1).

AP distribution among males was as follows: RAS 7.3%, RL 4.2%, RP 4.2%, RPS 31.2%, LL 24%, LP 9.4%, LAL 4.2%,RPL 2.1%, LPL 5.2 %, LPS 3.1%, LAS 3.1%, RAL 1%, and LAL with LPS 1%, and as follows among females: RAS 9.5%, RL 2.4%, RP 9.5%, RPS 16.7%, LL 35.7%, LP 14.3%, LAL 0%,RPL 4.8%, LPL 4.8%, LPS 0%, LAS 2.4%, RAL 0%, and LAL with LPS 0% (Table 2).

**Table 2:** The distribution of the accessory pathway locations according to gender left anteroseptal (LAS), left anterolateral (LAL), left lateral (LL), left posterolateral (LPL), left posterior (LP), left posteroseptal (LPS), right posteroseptal (RPS), right posterior (RP), right posterolateral (RPL), right lateral (RL), right anterolateral (RAL), right anteroseptal (RAS)

Location	Male	Female	Total
RAS	7 (7.3%)	4 (9.5%)	11 (8%)
RL	4 (4.2%)	1 (2.4%)	5 (3.6%)
RP	4 (4.2%)	4 (9.5%)	8 (5.8%)
RPS	30 (31.2%)	7 (16.7%)	37 (26.8%)
LL	24 (24%)	15 (35.7%)	38 (27.5%)
LP	9 (9.4%)	6 (14.3%)	15 (10.8%)
LAL	4 (4.2%)	0	4 (2.9%)
RPL	2 (2.1%)	2 (4.8%)	4 (2.9%)
LPL	5 (5.2%)	2 (4.8%)	7 (5%)
LPS	3 (3.1%)	0	3 (2.1%)
LAS	3 (3.1%)	1 (2.4%)	4 (2.9%)
RAL	1 (1%)	0	1 (0.7%)
LAL+LPS	1 (1%)	0	1 (0.7%)
Total	97 (100%)	42 (100%)	139 (100%)

**Figure 2:**
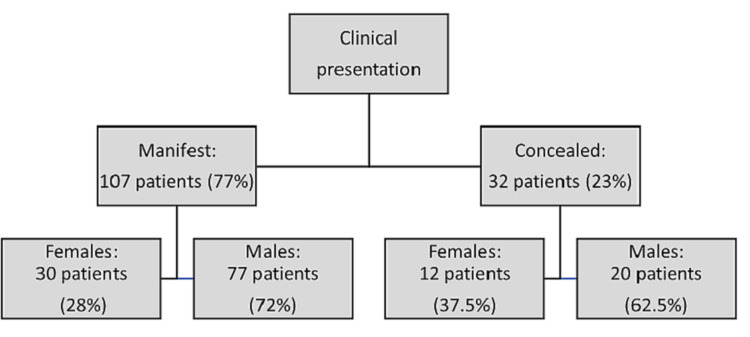
The clinical presentations of accessory pathways according to gender

Concerning clinical presentation, 107 (77%) of the pathways were manifest and 32 (23%) were concealed. The distribution among males and females is shown in Figure 2. Although males were more prevalent in both groups, the difference was statistically nonsignificant with a p-value of 0.38 (Figure 2).

## Discussion

Accessory pathways (APs) vary in anatomical locations and may be capable of conduction from atria to ventricles in an anterograde form, only in a retrograde form from ventricles to atria, or may have bidirectional conduction properties as in the classical Wolff-Parkinson-White syndrome [8]. Electrophysiologic studies and mapping have shown that accessory atrioventricular (AV) pathways may be located anywhere along the AV ring (groove) or in the septum. Catheter ablation has become the primary therapy to treat those accessory pathways [6-7].

In a retrospective cross-sectional study between January 2002 and December 2016, described by Jamal et al., most APs were identified in males (62.4%). Overall, left accessory pathways were predominant in the studied population (54.1%), with a mean age of diagnosis of 35.36 ±12.44 years [9]. In his observational study, Ferro et al. found that APs were more prevalent in males (52.6%), and the mean age of diagnosis was 31.2 ±13.8 years [10]. Those results are consistent with those concluded in this study. The majority of the 139 patients diagnosed with APs were males (69.8%) and 30.2% were females (p-value 0.04). The mean age was 32.2±13.5 years, which is in the usual age range for diagnosing accessory pathways [9-10]. Most of the patients were diagnosed between the ages of 20 and 29 years (Table 1).

As regards AP locations, different studies described different distributions. In a study by Ardakani et al., among 178 diagnosed with Wolff-Parkinson-White syndrome, the most common location was left lateral (LL), representing 39.3% of the overall cases studied [11]. In the study described by Birati et al., most APs in the 804 patients enrolled had a left free wall location (57.8%) [12]. Similarly, this study showed that most patients were diagnosed with LL APs (Table 2). However, only 27.5% of the patients were diagnosed with LL, which is less than that observed in previously conducted studies [11]. Besides, the population studied had a higher rate of RPS (26.8%) (Table 2). According to the literature, 25% of APs are posteroseptal in distribution [13]. This includes not only RPS but also LPS pathways. Furthermore, in the study conducted by Wen et al., among 652 patients, only 45 (7%) were found to have RPS AP [14].

Overall, most of the 139 patients studied had left-sided accessory pathways (52.5%) consistent with some literature estimates [11-12]. As regards gender, different studies yielded different results. Hsu et al. reported that females had 2.8-fold greater odds of having a right annular AP than males [15]. On the contrary, this study, similar to that described by Birati et al. [12], showed that left pathways were predominant in both males (51%) and females (57%) (Figure 1). Moreover, the most common accessory pathway among the males was right posteroseptal (RPS) with a percentage of 31.2%, followed by LL (24%). The females' most common pathways turned out to be LL at 35.7%, followed by RPS at 16.6% (Table 2). Again, this differs from a previously published paper showing that the most common accessory pathway among the males was LA, and the most common among females was right posteroseptal (RPS) [9]. Those different results between studies might signify that the pathways' locations are affected by race and gender.

As mentioned previously, some accessory pathways conduct only in a retrograde direction, from ventricles to atria; those are called concealed pathways because they do not pre-excite the ventricles during normal sinus rhythm and the surface electrocardiogram (EKG) appears normal [8]. It is estimated that 17% to 37% of APs are concealed [16]. In this study, 23% of the pathways were concealed, and 77% were manifest (Figure 2). Despite the predominance of males in both clinical groups, the difference among gender was statistically nonsignificant (p-value 0.38).

## Conclusions

In the population studied, left-sided accessory pathways were the most common. Males more commonly had RPS accessory pathways while LL pathways were predominant among females. Such results differ from those of studies conducted in other countries. These findings support the possible role of gender in the pathogenesis of accessory pathway formation.
